# Fluorescence Polarization Imaging of Methylene Blue Facilitates Quantitative Detection of Thyroid Cancer in Single Cells

**DOI:** 10.3390/cancers14051339

**Published:** 2022-03-05

**Authors:** Peter R. Jermain, Andrew H. Fischer, Lija Joseph, Alona Muzikansky, Anna N. Yaroslavsky

**Affiliations:** 1 Advanced Biophotonics Laboratory, University of Massachusetts Lowell, Lowell, MA 01854, USA; pjermain@mgh.harvard.edu; 2 Department of Radiation Oncology, Massachusetts General Hospital, Boston, MA 02114, USA; 3 Department of Pathology, University of Massachusetts Medical School, Worcester, MA 01655, USA; andrew.fischer@umassmemorial.org; 4 Department of Pathology and Laboratory Medicine, Lowell General Hospital, Lowell, MA 01854, USA; lija.joseph@lowellgeneral.org; 5 Biostatistics Center, Massachusetts General Hospital, Boston, MA 02114, USA; amuzikansky@mgh.harvard.edu; 6 Department of Dermatology, Massachusetts General Hospital, Boston, MA 02114, USA

**Keywords:** thyroid cancer, cytopathology, methylene blue, fluorescence polarization, quantitative imaging

## Abstract

**Simple Summary:**

Accurate diagnosis of thyroid fine-needle aspiration cytology is a significant clinical challenge. A method to detect thyroid cancer at the cellular level would be invaluable to reduce diagnostic uncertainty and improve clinical decision making. We studied the ability of confocal fluorescence polarization imaging of an exogenous fluorophore, methylene blue, to provide quantitative discrimination of cancerous cells in human samples. Our results indicate that fluorescence polarization imaging provides a reliable biomarker of thyroid cancer and holds the potential to shift the paradigm of cellular level cancer diagnosis from subjective visual assessment to objective measurement.

**Abstract:**

Background: Diagnostic accuracy of the standard of care fine-needle aspiration cytology (FNAC) remains a significant problem in thyroid oncology. Therefore, a robust and accurate method for reducing uncertainty of cytopathological evaluation would be invaluable. Methods: In this double-blind study, we employed fluorescence emission and quantitative fluorescence polarization (Fpol) confocal imaging for sorting thyroid cells into benign/malignant categories. Samples were collected from malignant tumors, benign nodules, and normal thyroid epithelial tissues. Results: A total of 32 samples, including 12 from cytologically indeterminate categories, were stained using aqueous methylene blue (MB) solution, imaged, and analyzed. Fluorescence emission images yielded diagnostically relevant information on cytomorphology. Significantly higher MB Fpol was measured in thyroid cancer as compared to benign and normal cells. The results obtained from 12 indeterminate samples revealed that MB Fpol accurately differentiated benign and malignant thyroid nodules. Conclusions: The developed imaging approach holds the potential to provide an accurate and objective biomarker for thyroid cancer, improve diagnostic accuracy of cytopathology, and decrease the number of lobectomy and near-total thyroidectomy procedures.

## 1. Introduction

Epidemiological studies indicate that overall prevalence of thyroid nodules is between 40 and 71% in adults [1,2]. Even though the vast majority (~90%) of these tumors are benign proliferations [3], the incidence of thyroid cancer is rising [4] and more than 44,000 cases are expected to be diagnosed in 2021 in the United States [5]. Therefore, early reliable discrimination of cancerous and benign lesions is of primary importance for proper thyroid cancer management.

Fine-needle aspiration cytology (FNAC) is the standard of care for preoperative evaluation of thyroid nodules that are suspicious for malignancy [6]. The Bethesda System for Reporting Thyroid Cytopathology (TBSRTC) is a standardized classification scheme used to group thyroid cytology specimens into one of six diagnostic categories: (I) non-diagnostic/unsatisfactory (ND/UNS), (II) benign, (III) atypia of undetermined significance/follicular lesion of undetermined significance (AUS/FLUS), (IV) follicular neoplasm/suspicious for a follicular neoplasm (FN/SFN), (V) suspicious for malignancy (SFM), and (VI) malignant [7]. While FNAC can provide a conclusive diagnosis for benign (category II) and malignant (category VI) thyroid nodules [8,9], overall diagnostic accuracy ranges from 60.2 to 68.8% when including aspirates within the indeterminate categories III–V [10,11,12,13,14]. The lesions of indeterminate categories account for approximately 30% of all thyroid nodules that are biopsied [15,16]. Consequently, definitive diagnosis requires histological assessment of the nodule following lobectomy or near-total thyroidectomy, committing the patient to a lifetime regimen of hormone-replacement therapy [17].

During the last decade, molecular testing has been increasingly utilized to reduce diagnostic uncertainty and guide therapeutic decision-making following indeterminate cytological findings [18]. The tests identify specific gene abnormalities associated with follicular cell-derived thyroid cancers [19,20,21,22]. Validation data have demonstrated that genomic profiling can result in fewer unnecessary diagnostic surgeries [22]. However, accurate molecular testing methods are limited to highly specialized laboratories, are expensive, and have long turnaround times [18,22]. Moreover, several reports have questioned diagnostic accuracy and repeatability of results. For example, in multi-center clinical trials the NPV of Afirma Gene Expression Classifier (GEC) (Veracyte, San Francisco, CA, USA) was 69% [23], whereas PPV of ThyroSeq v2 (Sonic Healthcare USA, Austin, TX, USA) was as low as 22–43% [24]. A robust, expedient, low-cost method that could provide an accurate quantitative marker for cancer would be invaluable for reducing diagnostic uncertainty of thyroid FNAC.

Methylene blue (MB) is cytological stain that has demonstrated affinity to cancer [25,26]. It is approved for several clinical applications by the United States Food and Drug Administration [27,28] and there is a progressively increasing number of publications focused on MB in the context of thyroid oncology-based problems [29,30,31,32]. Recent reports have demonstrated that fluorescence polarization (Fpol) of MB is significantly elevated in cancerous human breast ductal carcinoma and glioblastoma cell lines [33,34], and in malignant renal FNAC specimens [35]. Fluorescence polarization is a well-established optical technique that characterizes polarization state of fluorescence emission [36]. Unlike fluorescence emission, Fpol is not altered by the absorption or scattering properties of surrounding tissues and it does not require precise staining or illumination protocols. Moreover, it yields quantitative results in real time. In this study, we conducted the first evaluation of MB Fpol in thyroid cells obtained from clinical pathologically diverse thyroid tissues.

## 2. Materials and Methods

### 2.1. Study Design

Samples were obtained from excess, deidentified thyroid tissues following thyroid lobectomy or total thyroidectomy at the University of Massachusetts Medical Center (UMMC) in Worcester, MA, USA, or Lowell General Hospital (LGH) in Lowell, MA, USA. Study specimens were transported to the Advanced Biophotonics Laboratory (ABL) in Lowell, MA, USA, where fluorescence emission and polarization confocal images were acquired and Fpol values of single thyroid cells were processed. After imaging, cell viability was assessed. The results of optical imaging were evaluated against the gold standard of clinical H&E histopathology. This was a double-blind study; specifically, researchers at ABL were blinded to the results of histopathological analysis at the time of imaging and data analysis and the study pathologists were blinded to the results of the optical imaging at the time of histopathological analysis. The results of the study were statistically evaluated using a mixed-effects linear model. Statistical analysis was performed on all the samples and, separately, on the samples obtained from cytologically indeterminate categories.

### 2.2. Study Samples

Sample information is summarized in Table 1, columns 1–4. The specimens were collected from malignant tumors (medullary thyroid carcinoma, MTC; papillary thyroid carcinoma, PTC; follicular thyroid carcinoma, FTC), benign nodules (follicular thyroid adenoma, FTA; multinodular goiter, MNG), and normal thyroid epithelial tissues. Fresh tissue in the nodule or adjacent normal thyroid tissue was scraped by one of the study pathologists using a sterile surgical blade (4-122, Miltex, Inc., Plainsboro, NJ, USA) to collect cells for optical imaging/analysis. The cells were deposited in 1.5 mL tubes filled with Leibovitz’s L-15 growth medium (21083-027, ThermoFisher Scientific, Waltham, MA, USA) and transported to the ABL of the University of Massachusetts Lowell. The cells were kept in L-15 medium during transportation and arrived at ABL within 1 h following collection. The cells were plated in 4-well glass bottom imaging dishes (D35C4-20-1.5-N, Cellvis, Mountain View, CA, USA) that were treated for 1 h with 0.01% Poly-L-Lysine solution (P4707-50 mL, Millipore Sigma, Burlington, MA, USA) to enhance cell adhesion. Prior to imaging the cells were kept at 37 °C and relative humidity of 95%. The cells attached to the glass surface within 12 h, as confirmed using a Zeiss Primovert microscope (Carl Zeiss Microscopy, Oberkochen, Germany). To lyse erythrocytes, the cell monolayers were incubated with 0.5 mL of RBC Lysis Buffer (J62990, Alfa Aesar, Haverhill, MA, USA) for 5 min. The RBC Lysis Buffer was rinsed with 1X Phosphate Buffered Saline (PBS, J61917.AP, Alfa Aesar, Haverhill, MA, USA). Cells were then incubated with 156.3 µM (0.05 mg/mL) aqueous methylene blue solution (837519, 1% injection USP, McKesson Corporation, San Francisco, CA, USA) for 20 min. Following staining, the cell layers were washed 3 times with PBS to remove excess dye, and each well was filled with 0.3 mL L-15 medium.

Immediately after staining and rinsing, optical images of the samples were acquired using an in-house built confocal microscope. During imaging, the cells were kept at the ambient room temperature of 18 °C.

### 2.3. Multimodal Optical Imaging

**Figure 1:**
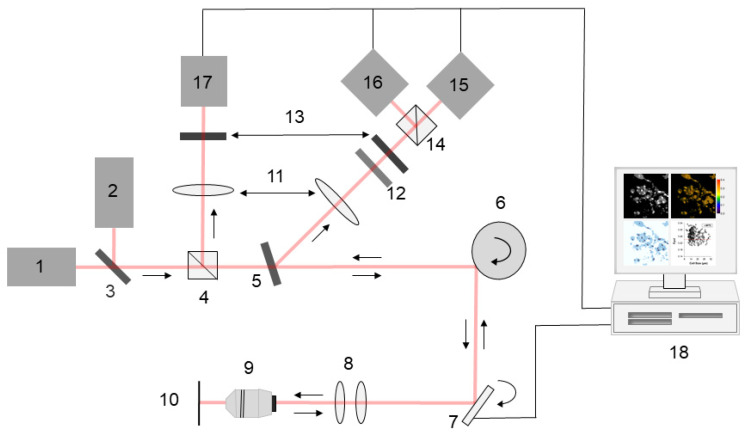
Multimodal confocal imaging system. (1) 642 nm laser, (2) 532 nm laser, (3) 45° dichroic mirror, (4) nonpolarizing beam splitter, (5) 12° dichroic mirror, (6) polygon mirror, (7) galvanometer mirror, (8) microscopic optics, (9) objective lens, (10) sample plane, (11) focusing lens, (12) fluorescence filter, (13) confocal pinhole, (14) polarizing beam splitter, (15) co-polarized fluorescence photomultiplier tube (PMT), (16) cross-polarized fluorescence PMT, (17) reflectance PMT, (18) computer. Red line traces the optical path.

The multi-channel confocal microscope displayed in Figure 1 was used to acquire reflectance, fluorescence emission, and polarization images.

Illumination was provided by two linearly polarized laser sources. The 642 nm diode laser (L46425-120-TE, Micro Laser Systems, Garden Grove, CA, USA) was used for reflectance, fluorescence emission, and fluorescence polarization imaging. Reflectance imaging with the 532 nm laser (BWI-532-5E, B&W Tek, Newark, DE, USA) guided sample positioning to avoid photobleaching of methylene blue. Beams were combined using a 45° dichroic mirror (Iridian Spectral Technologies, Ottawa, ON, Canada). A polygon mirror (DT-36-290-025, Lincoln Laser, Phoenix, AZ, USA) and galvanometric mirror (General Scanning Inc., Billerica, MA, USA) were used to scan the incident beam across x- and y-directions, respectively. Scanning was performed at the rate of 7 frames per second. A 63X/NA 1.4 oil immersion objective lens (440762-9904, Carl Zeiss, Oberkochen, Germany) focused light onto the sample. Light remitted from the sample was collected by the objective lens and de-scanned. Fluorescence signal was separated from elastically scattered light using a 12° dichroic mirror (Iridian Spectral Technologies, Ottawa, ON, Canada), transmitted through a 690 nm bandpass filter (Chroma, Bellows Falls, VT, USA) with full width at half maximum of 20 nm, then focused by a lens onto a 100 μm diameter pinhole (56-283, Edmund Optics, Barrington, NJ, USA). Orthogonal components of the fluorescence emission were separated by a polarizing beam splitter (MSBTS-12-45, Karl Lambrecht Corporation, Chicago, IL, USA) and registered by two photomultiplier tubes (PMT) (R9110, Hamamatsu Photonics, Shizuoka Prefecture, Japan). Elastically scattered light was reflected by a non-polarizing beam splitter (Thorlabs, Newton, NJ, USA) and focused by a lens onto a 100 µm pinhole positioned in front of the third PMT. Signals were recorded as 8-bit grayscale images using VivaScan software (Caliber ID, Andover, MA, USA). The system yielded lateral resolution better than 0.9 μm and axial resolution of 2 μm, and field of view of 205 µm × 205 µm.

### 2.4. Image Processing

Fluorescence emission images were processed in ImageJ (available from the National Institutes of Health). Co-polarized and cross-polarized fluorescence images were thresholded to remove background (pixel values below 3) and saturated pixels with values greater than 254. The co-polarized and cross-polarized fluorescence images were averaged over 3 frames, then used to generate the fluorescence emission image. We digitally stained the fluorescence emission images based on pixel intensity to mimic Papanicolaou stain, with cell nuclei blue-purple and cytoplasm blue-green in color.

Fpol image processing was performed using MetaMorph imaging software (version 7.10.2, Molecular Devices, Sunnyvale, CA, USA). Cells were manually segmented. Pixel values of each cell in averaged co-polarized ($F_{co}$) and cross-polarized ($F_{cross}$) fluorescence images were calculated, and Fpol was determined [36]:

(1) $$Fpol=(F_{co}-G\times F_{cross})/\left(F_{co}+G\times F_{cross}\right)$$

where $F_{co}$ and $F_{cross}$ are fluorescence emissions polarized in the plane parallel and perpendicular to that of the incident light, respectively. G is the calibration factor of the imaging system and determined to equal 0.75 using the methodology derived from Seigel et al. [37].

The Fpol of each sample was calculated by averaging Fpol data over all the cells in the sample.

### 2.5. Trypan Blue Exclusion Test

Cell viability after imaging was evaluated using a Trypan Blue (TB) exclusion test [38]. The cell monolayers were stained with TB solution (15250061, 0.4%, ThermoFisher Scientific, Waltham, MA, USA) for 1 min, then washed three times with PBS to remove excess dye. Next, the cells were imaged and counted using a brightfield microscope (Primovert, Carl Zeiss Microscopy, Oberkochen, Germany) equipped with a plan apochromat 20X/NA 0.3 air immersion objective lens (415500-1614, Carl Zeiss Microscopy, Oberkochen, Germany). Living cells were stained by MB only and appeared blue. Dead cells were stained by MB and TB and appeared purple. Example images of MB- and TB-stained cells are presented in Figure S1. The viability of the sample was calculated using Equation (2):

(2) $$Viability=(N_{Viable}/N_{Non\text{-}viable})\times 100$$

where $N_{Viable}$ and $N_{Non\text{-}viable}$ represent the number of viable cells and non-viable cells in the sample, respectively.

### 2.6. Statistical Analysis

The thyroid samples were organized in 3 diagnostic groups (malignant—MTC, PTC, and FTC samples; benign—FTA and MNG samples; normal—normal samples) and 5 histological groups (PTC, FTC, FTA, MNG, normal) for statistical evaluation. In addition, 12 indeterminate samples were organized in 4 groups (TBSRTC III-benign, TBSRTC III-malignant, TBSRTC IV-malignant, TBSRTC V-malignant) for statistical analysis. Least squares estimates of mean Fpol, and corresponding standard error were obtained for each group. A mixed-effects linear model that accounted for fixed effects and random effects [39] was implemented to evaluate statistical significance of differences between cancerous and non-cancerous samples with *p* < 0.05 considered significant. At least two samples per group were analyzed; therefore, the single MTC sample was excluded from the mixed-effects model analysis of histological groups.

## 3. Results

In total, we imaged and analyzed 32 samples, including 19 from UMMC and 13 from LGH. The samples were obtained from 21 patients and contained 3521 cells. The results of imaging and clinical histopathological analysis are summarized in Table 1, columns 3–8. Overall, 20 samples were from thyroid nodules and 12 samples were from normal thyroid glandular tissue. According to postoperative clinical histopathology, there were 13 malignant cases including 1 MTC, 9 PTC, and 3 FTC. There were 7 benign cases including 3 FTA and 4 MNG as well as 12 normal thyroid specimens. Cell viability following the imaging experiments was greater than 91%.

### 3.1. MB Fpol Is Significantly Elevated in Thyroid Cancer

**Figure 2:**
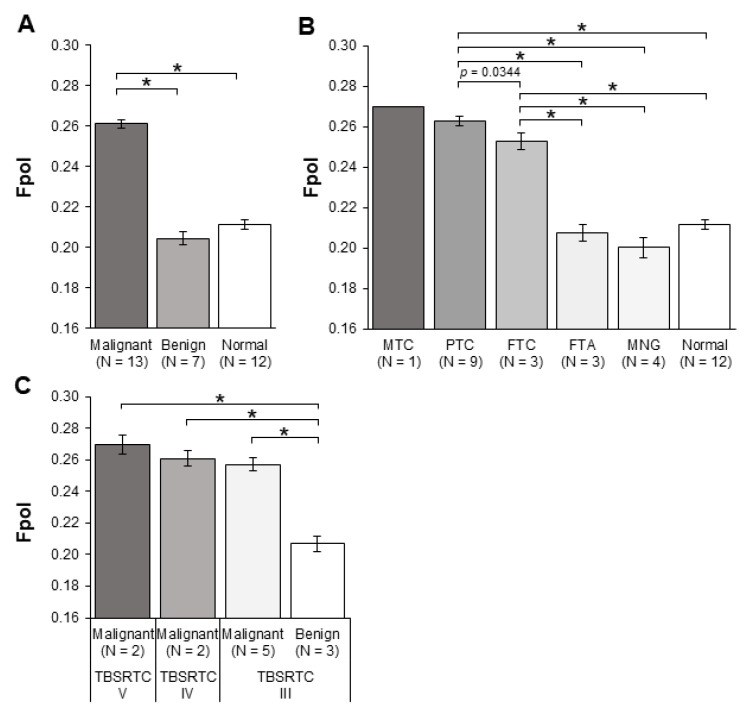
MB Fpol of thyroid samples. (**A**) Average MB Fpol of malignant, benign, and normal samples; (**B**) average MB Fpol of MTC, PTC, FTC, FTA, MNG, and normal samples; (**C**) average MB Fpol of indeterminate samples. Error bars represent standard errors calculated over all samples in the respective group (not determined for single MTC case). N = number of samples. * *p* < 0.0001.

The results of quantitative MB Fpol analysis, presented in Figure 2, reveal that cancerous samples exhibited higher average MB Fpol as compared to benign tumor or normal samples. To summarize, Figure 2A shows that average Fpol value of all malignant cases grouped together (MTC, PTC, and FTC) was 0.261 ± 0.002, whereas that of all benign tumor samples (FTA and MNG) was 0.205 ± 0.003. The normal samples had an average Fpol value of 0.211 ± 0.002. Statistical analysis demonstrated that differences in MB Fpol between malignant and benign/normal groups were highly significant (*p* < 0.0001). As shown in Figure 2B, for the thyroid carcinoma subtypes, PTC and FTC average Fpol values were equal to 0.263 ± 0.002 and 0.253 ± 0.004, respectively. Statistical analysis revealed significant differences between PTC and FTC (*p* = 0.0344). Additionally, we have analyzed one sample of MTC, which exhibited the second highest average Fpol of 0.270 ± 0.028. In benign nodules, average Fpol was 0.207 ± 0.004 for the FTA cases and 0.200 ± 0.005 for MNG cases. Fpol of PTC and FTC were significantly elevated (*p* < 0.0001) relative to FTA, MNG, and normal for all cancerous–noncancerous sample comparisons.

### 3.2. MB Fpol Accurately Discriminates Cytologically Indeterminate Nodules

Twelve of the tumor samples (1719 cells) were acquired from lesions that yielded indeterminate results on clinical FNAC. This included 8 category III, 2 category IV, and 2 category V nodules. Category III samples included 3 PTC (592 cells), 2 FTC (285), and 3 FTA (408 cells). Category IV samples included 1 PTC (84 cells) and 1 FTC (89 cells), whereas in category V there were 2 PTC samples (261 cells). Figure 2C presents quantitative analysis of the indeterminate cases. The average Fpol of cancer samples was 0.270 ± 0.006 in category V and 0.261 ± 0.005 in category IV. Average Fpol of malignant and benign tumors in category III were 0.257 ± 0.003 and 0.207 ± 0.004, respectively. The results demonstrate significantly higher (*p* < 0.0001) MB Fpol in cancerous versus noncancerous indeterminate nodules for all malignant–benign sample comparisons.

### 3.3. Fpol Images Provide Quantitative Cellular Level Contrast for Cancer Detection

**Figure 3:**
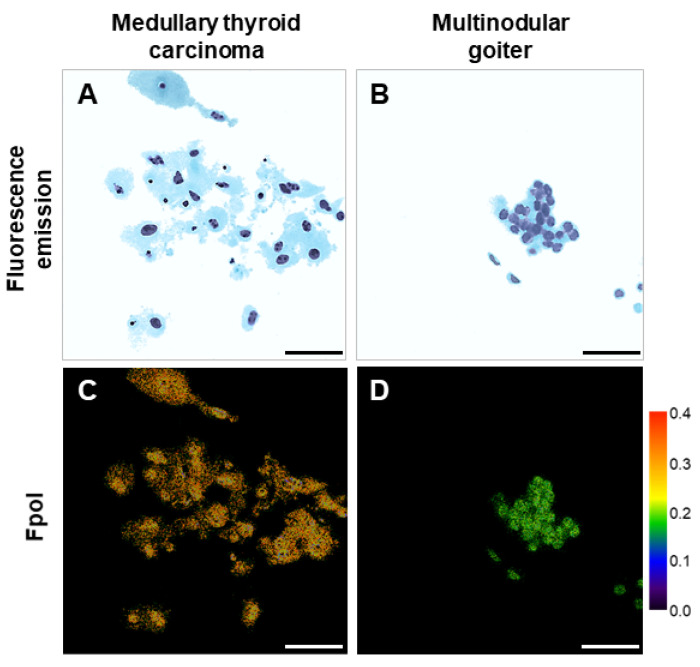
Malignant and benign samples. Example MB fluorescence emission images for (**A**) MTC sample (subject 1) and (**B**) MNG sample (subject 17); (**C**,**D**) corresponding pseudo-colored MB Fpol images. Scale bar: 50 µm.

In Figure 3, representative images of cancerous and benign cells are shown. The fluorescence emission images in Figure 3A,B were digitally stained to mimic Papanicolaou stain and display cytomorphology. Respective grayscale fluorescence emission images are presented in Figures S2 and S3. The corresponding pseudo-colored MB Fpol images (Figure 3C,D) provide a quantitative assessment. The Fpol scale located next to the image ranges from 0.0 (black) to 0.40 (red). Figure 3A shows loose clusters of large MTC cells (subject 1) with round nuclei obtained from a TBSRTC VI nodule. Figure 3B shows MNG cells (subject 17), including bland thyrocytes organized in a flat sheet, obtained from a TBSRTC II nodule. H&E histopathology confirmed the diagnoses of medullary thyroid carcinoma and multinodular goiter. The quantitative images demonstrate elevated MB Fpol values in the MTC cells (Figure 3C) relative to MNG cells (Figure 3D). Fpol values of MTC and MNG cells shown in Figure 3 ranged between 0.231–0.315 and 0.181–0.209, respectively. Average Fpol of the MTC sample was 0.270 ± 0.028, whereas that of the MNG sample was 0.207 ± 0.026.

**Figure 4:**
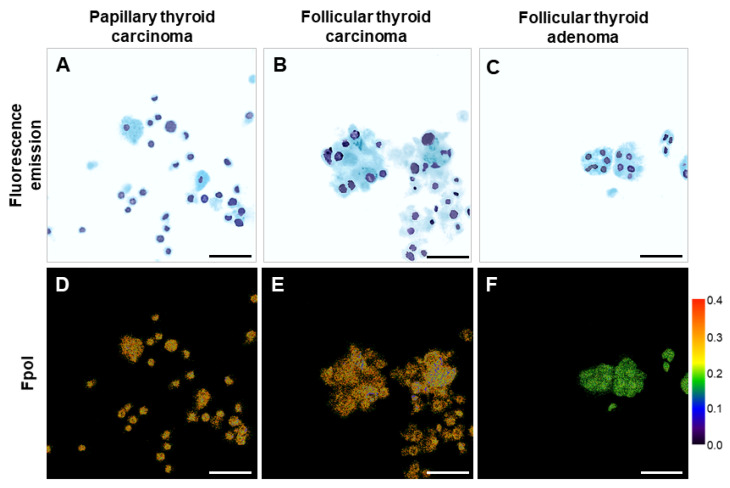
Indeterminate samples. Example MB fluorescence emission images for (**A**) PTC sample (subject 9), (**B**) FTC sample (subject 12), and (**C**) FTA sample (subject 14); (**D**–**F**) corresponding pseudo-colored MB Fpol images. Scale bar: 50 µm.

Figure 4 presents example fluorescence emission and polarization images of indeterminate thyroid nodules. Figure 4A–C display example digitally stained MB fluorescence emission images of PTC cells (subject 9), FTC cells (subject 12), and FTA cells (subject 14), respectively. The respective grayscale images are shown in Supplementary Figures S4–S6. Each sample was TBSRTC III on cytopathology and had diagnosis confirmed by histopathology. Cytologic features of the PTC cells are discernible in Figure 4A. Malignant FTC cells (Figure 4B) and benign FTA cells (Figure 4C) display similar morphology including microfollicles with nuclear overlapping and crowding. Corresponding Fpol images of the cells are shown in Figure 4D (PTC), Figure 4E (FTC), and Figure 4F (FTA). Fluorescence polarization of MB is visibly higher in cancer. In comparison, Fpol values for the cells shown ranged from 0.243–0.310 (PTC), 0.228–0.310 (FTC), and 0.165–0.202 (FTA). Average Fpol of the samples were 0.259 ± 0.018, 0.250 ± 0.015, and 0.215 ± 0.018 for PTC, FTC, and FTA, respectively. Enhanced contrast from MB Fpol is particularly important for the follicular lesions (Figure 4E,F). Follicular carcinoma and adenoma cannot be distinguished at the cellular level using current cytologic criteria. Instead, the diagnosis relies on histologic evidence of capsular invasion and/or vascular invasion to determine whether the tumor is malignant [6].

### 3.4. Cellular MB Fpol Values Are Not Patient Specific

**Figure 5:**
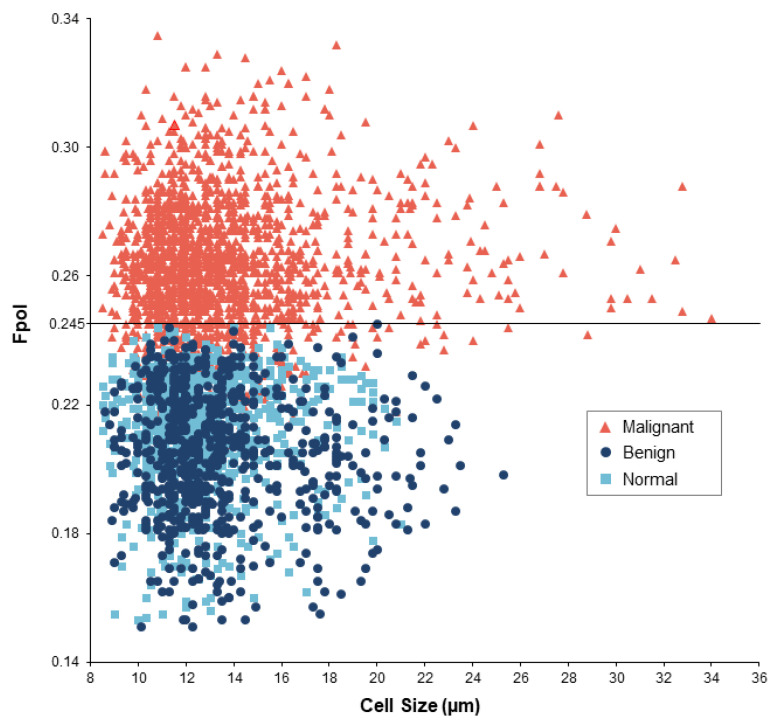
MB Fpol versus cell size for 3521 cells in 32 thyroid samples. Red triangles—malignant (1877 cells); dark blue circles—benign (724 cells); light blue squares—normal (920 cells). Black horizontal line represents Fpol value of 0.245.

Figure 5 presents a scatter plot displaying all imaged cells. Each cell is characterized by its size (x-axis) and Fpol value (y-axis). Cells from cancerous samples (MTC, PTCs, and FTCs) are shown as red triangles. Cells from benign samples (FTAs and MNGs) are shown as dark blue circles, and cells from normal thyroid samples are shown as light blue squares. The malignant cells exhibited higher Fpol values as compared to benign or normal cells. Specifically, malignant cell Fpol values ranged between 0.191 to 0.335, whereas benign and normal cell Fpol values ranged from 0.153 to 0.245 and 0.151 to 0.244, respectively. The plot reveals that there are no cells in the benign and normal samples with Fpol greater than 0.245 or 0.244, respectively. It can be appreciated that there is a pronounced overlap between MTC, PTC, and FTC cells obtained from different subjects. Similarly, there is noticeable overlap of FTA, MNG, and normal cells at lower Fpol values. This result indicates that cellular level MB Fpol values are not patient-specific and can separate cancer from benign tumors or normal.

## 4. Discussion

This is the first study utilizing exogenous Fpol of MB as a quantitative marker for thyroid cancer at the cellular level. Previous investigations of this technology were conducted with either breast or brain cultured cells [33,34]. However, cultured cells are identical and grown under controlled conditions. Therefore, they can serve only as a simplified model of the clinical cell aspirates. In this contribution, we imaged and analyzed thyroid cells obtained from the samples taken directly from the patients. These cells were heterogenous in terms of cell size, shape, and morphological classification (Figure 2, Figure 3, Figure 4 and Figure 5). Moreover, the identity of the cell lines was well known to researchers. In contrast, this was a double-blind study: the researchers were blinded to the results of histopathological evaluations during optical imaging and analysis, and study pathologists were blinded to the results of optical imaging during histopathological analysis.

In this pilot clinical study, postoperative clinical H&E histopathology was used to evaluate results of the Fpol imaging method. Comparison of the optical imaging results with histopathology established that MB Fpol exhibited by papillary carcinoma and follicular carcinoma samples was significantly higher (*p* < 0.0001) than that of normal or benign thyroid cells, including follicular adenoma and multinodular goiter (Figure 2). Additionally, one medullary thyroid carcinoma case was examined. It presented the second highest average Fpol of all cancer samples. As can be appreciated from Table 1 and Figure 5, there were no benign or normal cells with MB Fpol higher than 0.245. In comparison, 3–38% of cells in the cancerous samples yielded Fpol values lower than 0.245, which may be explained by the presence of non-cancerous cells in the tumor (e.g., lymphocytes). Data analysis revealed that MB Fpol is not patient specific. Thus, it may be possible to use Fpol of 0.245 as a threshold value for discriminating cancerous cells under controlled experimental conditions. However, it should be noted that this value was defined empirically based on the analysis of our limited set of thyroid nodules and may need adjustment as more data become available.

Evaluation of 12 indeterminate samples revealed that MB Fpol accurately discriminates benign and malignant thyroid nodules. Considering that cytopathological analysis, the current standard of care, does not provide reliable differentiation of category III–V lesions, this is one of the most important findings of the present study. Our results clearly demonstrate that Fpol values of TBSRTC III–V malignant cells are significantly higher (*p* < 0.0001) than those of benign.

Recently, molecular testing has been increasingly used for the diagnosis of indeterminate thyroid lesions [18]. Even though initial clinical studies demonstrated that various multi-gene panels may be suitable for detecting malignancy in thyroid FNAC [18,19,20], subsequent reports have questioned diagnostic accuracy and repeatability of results between institutions [23,24]. Therefore, utilization of molecular tests is recommended only in the context of a complete clinical workup including laboratory, cytology, and pathology results [40]. Moreover, as compared to MB Fpol imaging, molecular testing is expensive, time-consuming, and necessitates a dedicated FNAC pass without a guarantee of a definitive diagnosis [18,40].

Several imaging and spectroscopic approaches such as optical coherence tomography and microscopy [41], second harmonic generation microscopy [42], photoacoustic imaging [43], fluorescence-lifetime imaging microscopy [44], and Raman spectroscopy [45] are currently being investigated for discrimination of thyroid nodules. However, their downsides include high costs, slow data acquisition, as well as complex data processing and interpretation algorithms. In comparison, MB Fpol imaging provides rapid results that are robust, accurate, and easy to interpret. Specifically, Fpol imaging does not require sophisticated data processing algorithms or evaluation of cell morphology. Fpol images yield high contrast of cancer (Figure 3C,D and Figure 4D–F) and are immediately available. Single frame confocal imaging is similar to operating conventional microscopy systems used by pathologists and would require minimal re-training, while low-cost confocal systems have already been introduced for clinical applications [46]. Thus, Fpol imaging may offer advantages over the above-mentioned technologies in the context of clinical applications. In this study, image acquisition required approximately 10 min per sample. Manual cell segmentation was the most time-consuming data processing step requiring about 10–15 min per sample. To make data handling more efficient, in future, we plan to implement and use automated cell segmentation.

Currently, FNAC is based on the visual evaluation of cell morphology. This method is subjective and yields inconclusive results in ~15–30% of cases. The results of this study point toward clinical utility of exogenous MB Fpol as a quantitative biomarker for thyroid cancer to discriminate malignant samples from benign in TBSRTC III–V. A distinct advantage of the method is that quantitative polarization information can be evaluated together with morphological information yielded by fluorescence emission images. Digital staining of fluorescence emission images to mimic the color pattern of accepted cytological stains may aid clinical adoption by pathologists. In this work, the digital staining algorithm imitated Papanicolaou staining; however, it can be adjusted to yield any dye combination (e.g., H&E). Eventually, high resolution fluorescence emission images and high contrast quantitative images, both available in real time, may enable rapid on-site cancer detection. Moreover, the cells remained viable after imaging, which suggests the feasibility of an in vivo approach to cancer detection without actual tissue acquisition. Future studies should evaluate toxicity and safety considerations of potential in vivo applications.

## 5. Conclusions

This is the first study utilizing quantitative MB Fpol imaging for the detection of thyroid cancer at the cellular level. Its results indicate that MB Fpol may provide an accurate tool for assessment of cancer in thyroid FNAC samples. At a moderate cost, it could solve the significant clinical challenge of indeterminate thyroid cytology. Ultimately, this approach may yield a definitive diagnosis at the initial FNAC appointment, reduce costly molecular analysis, minimize unnecessary diagnostic surgery, and lessen damage to normal patient tissue.

## Figures and Tables

**Table 1:** Clinical information and experimental data for 32 thyroid samples obtained from 21 patients.

Subject	Site	Diagnosis	TBSRTC Category	Tumor Sample		Normal Sample	
				Number of Cells	Mean Fpol ± SD	Number of Cells	Mean Fpol ± SD
1	UMMC	MTC	VI	247	0.270 ± 0.028	47	0.209 ± 0.014
2	UMMC	PTC	VI	137	0.253 ± 0.016	84	0.211 ± 0.015
3	UMMC	PTC	VI	79	0.263 ± 0.019	72	0.219 ± 0.012
4	UMMC	PTC	VI	103	0.257 ± 0.014	52	0.220 ± 0.012
5	LGH	PTC	V	57	0.263 ± 0.015	125	0.215 ± 0.018
6	LGH	PTC	V	204	0.276 ± 0.019	-	-
7	LGH	PTC	IV	84	0.260 ± 0.014	-	-
8	LGH	PTC	III	128	0.268 ± 0.019	-	-
9	LGH	PTC	III	210	0.259 ± 0.018	-	-
10	LGH	PTC	III	254	0.263 ± 0.016	-	-
11	UMMC	FTC	IV	89	0.262 ± 0.019	25	0.221 ± 0.024
12	UMMC	FTC	III	94	0.250 ± 0.015	59	0.210 ± 0.012
13	LGH	FTC	III	191	0.246 ± 0.016	-	-
14	UMMC	FTA	III	134	0.215 ± 0.018	102	0.205 ± 0.021
15	UMMC	FTA	III	145	0.211 ± 0.021	90	0.216 ± 0.010
16	LGH	FTA	III	129	0.204 ± 0.025	86	0.219 ± 0.021
17	UMMC	MNG	II	70	0.207 ± 0.026	-	-
18-1	UMMC	MNG	II	50	0.196 ± 0.025	-	-
18-2	UMMC	MNG	II	51	0.210 ± 0.019	-	-
19	LGH	MNG	II	145	0.196 ± 0.015	-	-
20	LGH	Normal	-	-	-	100	0.221 ± 0.014
21	LGH	Normal	-	-	-	78	0.204 ± 0.043

UMMC—University of Massachusetts Medical Center; LGH—Lowell General Hospital; MTC—medullary thyroid carcinoma; PTC—papillary thyroid carcinoma; FTC—follicular thyroid carcinoma; FTA—follicular thyroid adenoma; MNG—multinodular goiter; SD—standard deviation.

## Data Availability

The experimental data are available from the corresponding author upon request.

## Supplementary Materials

The following supporting information can be downloaded at: https://www.mdpi.com/article/10.3390/cancers14051339/s1, Figure S1: Bright field images following cell viability test, Figure S2: MTC case (subject 1), Figure S3: MNG case (subject 17), Figure S4: PTC case (subject 9), Figure S5: FTC case (subject 12), Figure S6: FTA case (subject 14).
