# Supplementary material for: Fluorescence Polarization Imaging of Methylene Blue Facilitates Quantitative Detection of Thyroid Cancer in Single Cells
Source: Cancers (Basel). 2022 Mar 5;14(5):1339. doi: 10.3390/cancers14051339 (PMC8908998; doi:10.3390/cancers14051339)
Supplement: Supplementary file 1 [file cancers-14-01339-s001.zip › cancers-1585626-supplementary.pdf]

Supplementary Information for

Fluorescence Polarization Imaging of Methylene Blue Facilitates Quantitative Detection of Thyroid Cancer in Single Cells

Peter R. Jermain [1,2], Andrew H. Fischer [3], Lija Joseph [4], Alona Muzikansky [5] and Anna N. Yaroslavsky [1,6,*]

[1] Advanced Biophotonics Laboratory, University of Massachusetts Lowell, Lowell, MA 01854, USA; pjermain@mgh.harvard.edu
[2] Department of Radiation Oncology, Massachusetts General Hospital, Boston, MA 02114, USA
[3] Department of Pathology, University of Massachusetts Medical School, Worcester, MA 01655, USA; andrew.fischer@umassmemorial.org
[4] Department of Pathology and Laboratory Medicine, Lowell General Hospital, Lowell, MA 01854, USA; lija.joseph@lowellgeneral.org
[5] Biostatistics Center, Massachusetts General Hospital, Boston, MA 02114, USA; amuzikansky@mgh.harvard.edu
[6] Department of Dermatology, Massachusetts General Hospital, Boston, MA 02114, USA
* Correspondence: ayaroslavsky@mgh.harvard.edu; Tel.: +1-978-934-3766

This PDF file includes:

Figure S1: Bright field images following cell viability test.

Figure S2: MTC case (subject 1).

Figure S3: MNG case (subject 17).

Figure S4: PTC case (subject 9).

Figure S5: FTC case (subject 12).

Figure S6: FTA case (subject 14).

Viability Test

Cell viability after the imaging experiments was evaluated in 26 samples using a trypan blue exclusion test. Living cells in Supplementary Figure S1A are blue because they are stained by MB only. The dead cells in Supplementary Figure S1B are purple because they are stained by both MB and TB.

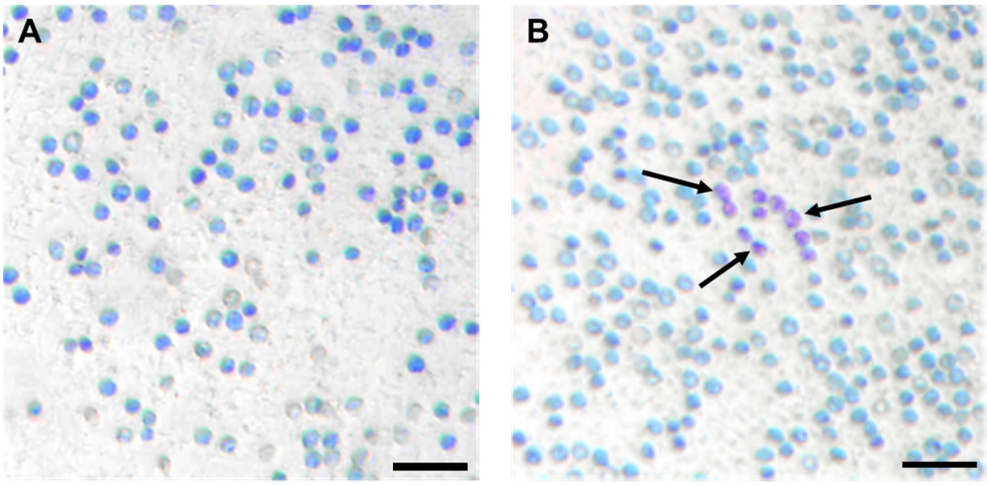

Figure S1. Bright field images following cell viability test. (A) Live cells stained with MB only (blue); **(B)** dead cells stained with MB and TB (purple). Solid black arrows: dead cells. Scale bar: 50 μm.

Digitally Stained and Grayscale Fluorescence Emission Images

Supplementary Figs. S2-S6 show a side-by-side comparison of digitally stained and grayscale fluorescence emission images. The digitally stained images mimic Papanicolaou stain to aid cytomorphological assessment by pathologists.

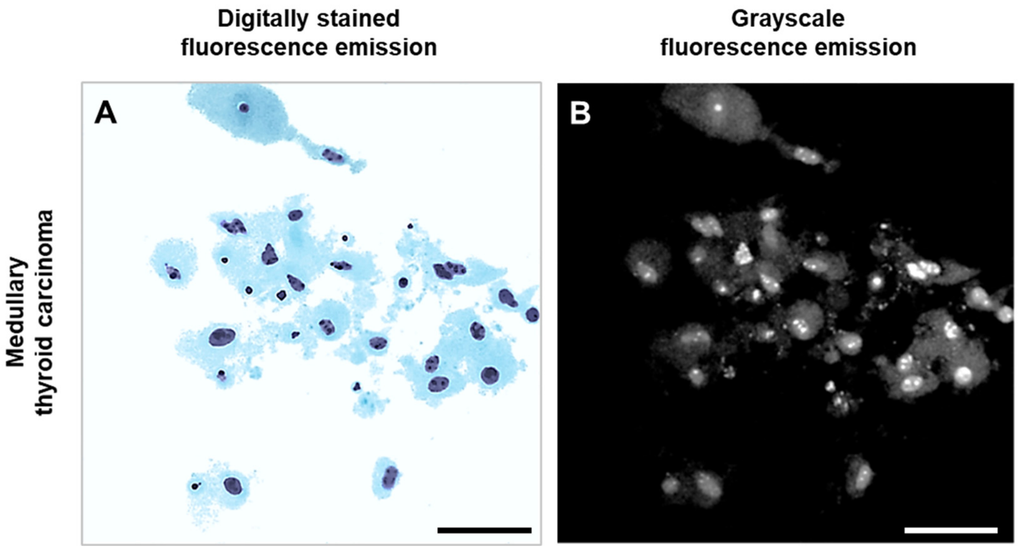

Figure S2. MTC case (subject 1). (A) Digitally stained fluorescence emission image; **(B)** grayscale fluorescence emission image. Scale bar: 50 μm.

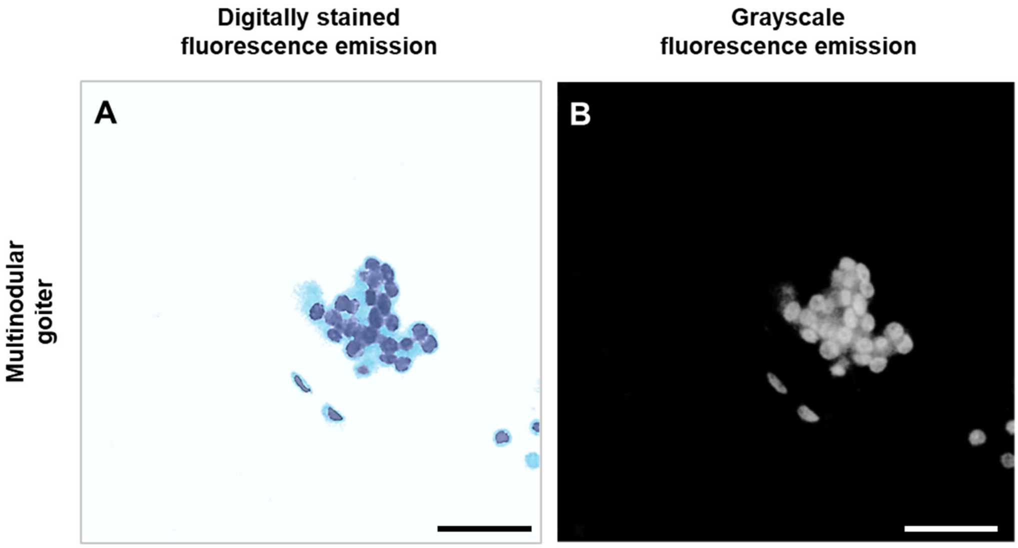

Figure S3. MNG case (subject 17). (A) Digitally stained fluorescence emission image; **(B)** grayscale fluorescence emission image. Scale bar: 50 μm.

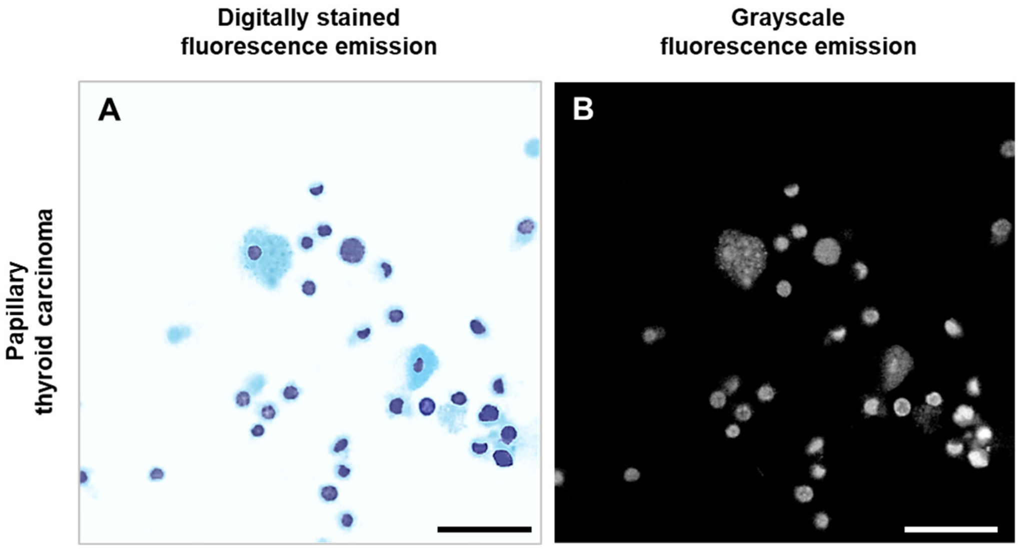

Figure S4. PTC case (subject 9). (A) Digitally stained fluorescence emission image; **(B)** grayscale fluorescence emission image. Scale bar: 50 μm.

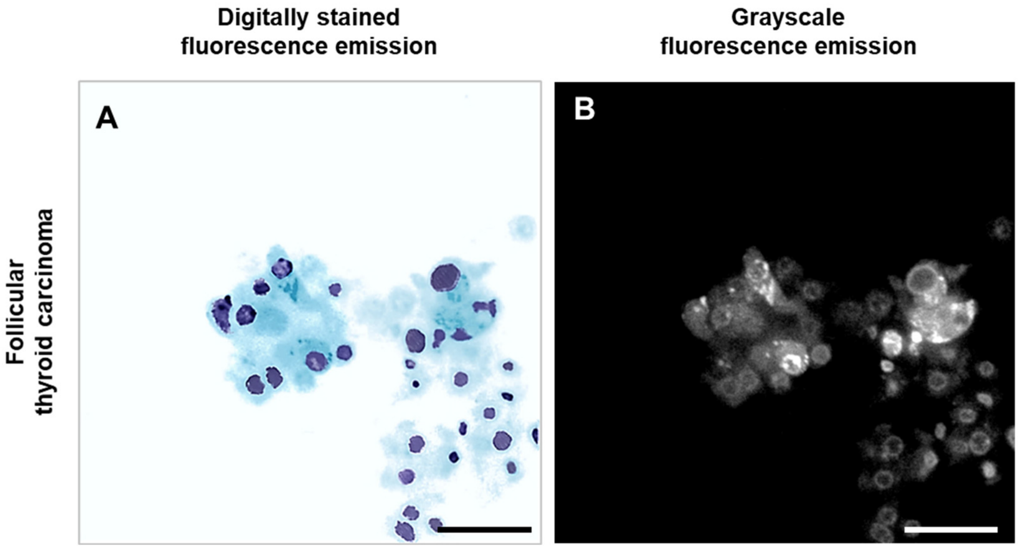

Figure S5. FTC case (subject 12). (A) Digitally stained fluorescence emission image; **(B)** grayscale fluorescence emission image. Scale bar: 50 μm.

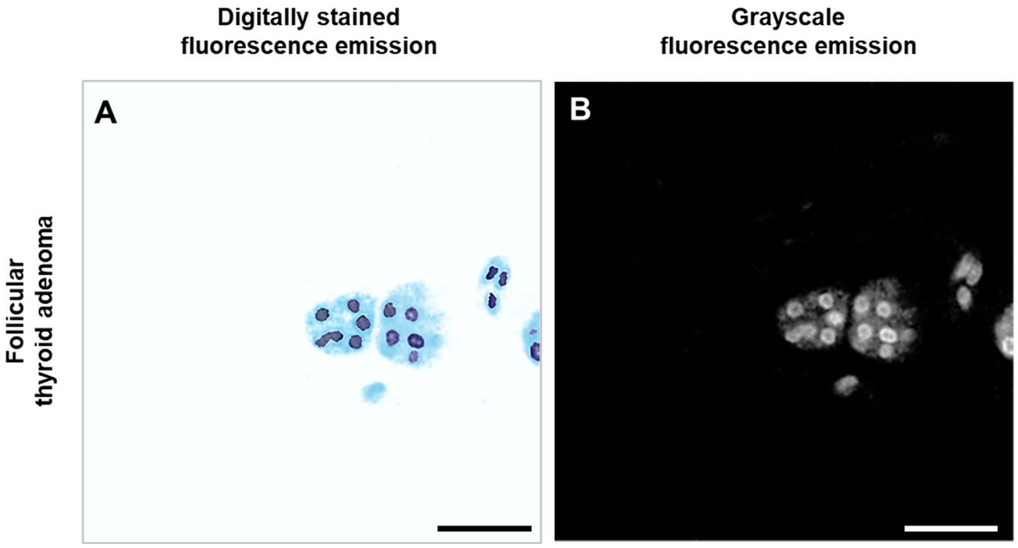

Figure S6. FTA case (subject 14). (A) Digitally stained fluorescence emission image; **(B)** grayscale fluorescence emission image. Scale bar: 50 µm.
